# ‘And then there was silence’: shaping practice through the experience of parents’ emotions

**DOI:** 10.1177/17449871231216057

**Published:** 2023-12-27

**Authors:** Sharon Levy

**Affiliations:** CPD Lead, Usher Institute, Edinburgh University, Edinburgh, UK

**Keywords:** emotional adjustment, parenting, prenatal care, prenatal education, qualitative research, spina bifida

## Abstract

**Background:**

Spina bifida, the most common Neural Tube Defect occurring around 28 days following conception, is often discovered in a routine ultrasound examination. Nurses offer continuous support for families who care for children with disabilities, associated with this diagnosis.

**Aim:**

To articulate and analyse parents’ recollection of the emotions they experienced when they were informed by professionals that their unborn baby may have neurological disabilities.

**Method:**

Qualitative study, using participatory visual narrative method, engaging parents caring for young children with Spina bifida.

**Results:**

The emotions parents described, surfacing at the point of diagnosis, and the crafted stories they shared, demonstrated the significance and the long-lasting impact of their experience. Disclosing sensitive information and the way it is shared has a significant role in shaping how families adjust to caring for disabled children.

**Conclusion:**

Role and care transitions of parents who choose to keep their babies, despite their impending disability, is a lengthy and complex process. Nurses should be aware of and informed by the origin story, to offer appropriate support throughout this period. Policies to promote integration with services delivered by ‘not for profit’ organisations will benefit family-focused and person-centred care.

## Introduction

Through advancement of medical technologies clinicians and expectant parents have nowadays a unique gaze with which to study and monitor the development of a foetus. An ultrasound appointment is a routine procedure that offers an opportunity to hear the heartbeat of the unborn baby, to get to know their sex as well as produce a first photo to be cherished. Yet, in a small percentage of cases, the examination reveals abnormal findings, which may require further testing or interventions and could induce parental distress, anxiety and anguish.

Spina bifida, the most common Neural Tube Defect (Blencowe et al., 2018), is occurring around 28 days following conception and often discovered in a routine ultrasound examination. According to the British Isles Network of Congenital Anomaly Registers (www.binocar.org) 1 in 1000 pregnancies in England and Wales are affected by spina bifida. In Scotland, however, there are more births with spina bifida, per head of population, than anywhere else in the United Kingdom according to the Scottish national database (www.sbhscotland.org.uk). Spina bifida is but one condition amongst a myriad of prenatal abnormalities but one that has a paucity of research examining in depth the impact the diagnosis has on families.

Whilst there is an increase in the number of babies with spina bifida that are born in Africa due to limited prenatal care, there is a marked decrease in live births in Europe and North America. Yet, recent research presented at the 2023 world congress on spina bifida research and care (Castillo et al., 2023) was dominated by papers from the United States. These focused on neurosurgical interventions in babies and on follow-up care for those with a shunt to release pressure on the brain. Others described research concerning multidisciplinary efforts to address complex clinical presentations by urology, orthopaedics and neuropsychology teams. Published research that seeks to give parents a voice is limited and often relates to the transition of young people from paediatric care to adult services (Betz et al., 2015; Ellison et al., 2022).

Meleis (2010) argued that midwives and nurses have a cardinal position in supporting a transition into a new role, that of a parent, which may be challenging. Indeed, there is an extensive body of literature that explores the skills, behaviours and attitudes that parents need to master as they transition to a caring role. Such a journey is much more complex if the new born has a life-limiting or a chronic ill health condition, coupled with intellectual or physical disability that requires extensive medical interventions, and long-term health and social care support. Considering the emotions involved in the moment of diagnostic disclosure, and how the impact shapes the caring transition thereafter, it is critical to have a better understanding of parental personal perspectives. Better knowledge should support the design of appropriate family and person-centred interventions that contribute to a positive overall outcome.

Engaging with families is fundamental to improving the quality and safety of care and person centredness is also an important mechanism to ensure the effectiveness and efficiency of the healthcare service (Carman et al., 2013; Park et al., 2018). The study reported here used ‘emotional touch points’ (Dewar et al., 2010) to capture data from parents residing across Scotland, who are caring for young children with neurological disabilities.

## Background

The literature concerning prenatal diagnosis and the impact it has on parents uses a myriad of descriptions such as: Emotional Confusion, Emotional Pain, Intense Grief, Psychological Distress, Anxiety, Depression, Loneliness, Prostration, Anger, Isolation, Anxiety, Insecurity, Guilt, Despair and so forth (Carlsson et al., 2017; Hodgson and McClaren, 2018; Lalor et al., 2007; Larsson et al., 2010). However, Freshwater (2004) suggests that the general population does not have sufficient emotional vocabulary to be able to name emotions. Yet, ‘*naming an emotion in itself can bring the individual closer to the affective element of it, as if by naming the emotion, we give the brain permission to access it*’ (Freshwater, 2004: 505). To ensure our participants were able to articulate their emotions and shape their ‘story’ accordingly we provided a range of pre-printed words of positive and negative feelings. These were found to stimulate, rather than constrain, personalised emotional expression (Nelson et al., 2017).

### Aim

The aim of the study was to articulate and interpret the caring experiences of parents of young children with spina bifida. We wanted to chart a transition pathway from the point of diagnosis to the present day, and to explore whether video recording software would give us a complementary gaze into the context of parental caring reality. Finally, we hoped to generate understanding and insights to inform and influence practice by advocating for the need for specific interventions and wider training.

### Design

We conducted a descriptive qualitative study that included participatory visual narrative methods consisting of Emotional Touch Points and video diaries. A touch point is an occasion where the person recalls being touched emotionally or cognitively (Dewar and Nolan, 2013). Recalling a subjective experience and associated emotions aid the crafting of a story that has the power to engage and affect the audience and offer an insight to the relationships between the care process and the story teller’s inner world (Dewar et al., 2010).

Participatory visual narrative, as was used in this study, may be a less familiar method than other participatory visual research tools such as photography in photovoice and drawing and mapping (Moletsane et al., 2017). Participants were invited to use a visual aid as a prompt to tell their story. Cards with a narrative that depict both positive and negative emotional words, as well as blank cards for participants to write their own emotional description, were used. This method actively engages participants in the research process, giving them the opportunity to shape the research process and content in ways that facilitate a sense of agency. The power of creativity and the opportunity to give participants a ‘voice’ (Mannay, 2015) may redress the unequal power relationships inherent in qualitative research (Thorogood, 2018). Moreover, using a participatory visual method offered a way for parents to express themselves and to communicate their experiences and perspectives in one of the most egalitarian means of communication – storytelling (Escobar, 2011).

### Sample/participants/process

Once the study gained ethical approval, through the University of Edinburgh School of Health in Social Science, the help and guidance of family support workers at a national charity, Spina Bifida Hydrocephalus Scotland (SBH Scotland), was enlisted. As the main Scottish hub for individuals and families affected by the condition, the association with the organisation was a key to successful recruitment of participants. Using the organisation communication channels that are used to share information with service users, we were able to identify a small sample to take part in the month-long study (*n* = 5). Participants had to agree to being involved in a face-to-face interview, as the first phase of the study, and to keep a video diary for a period of 1 month. We purchased a licence product that enabled a safe and secured upload and storage of mobile phone data, participants shared with us, offering a unique insight to their experience as carers.

The children had to be under the age of 5 years old and the carer had to have a good conduct of written and spoken English to participate in the study. The families we recruited were from the North, West and the East of Scotland and used different primary and tertiary healthcare service providers. The social and economic composition of each family unit was different but, in all cases, it was the mother who took the active part in the interview and the recording of video diaries.

Fathers were also welcome to participate and we aimed to be inclusive and target parents rather than focus exclusively on mothers. However, some fathers fall into a secondary role during pregnancy and feel like outsiders in the maternal domain following childbirth. Indeed, others have found various challenges in engaging fathers, including difficulties with scheduling and availability and differing parental perceptions concerning familial gender roles (Klein et al., 2022).

Interviews were scheduled to be taken at home at an arranged time convenient to the family. These were recorded once the process was explained and the consent form signed. The researcher described the emotional touchpoint method and proceeded to lay the printed cards whilst reading each word aloud. Words describing positive feelings were put to one side and words describing negative emotions were put on a different side and they were all visible throughout the interview. There were equal numbers of positive and negative words, and they were printed in a different font to distinguish the type of word they were. Participants were then asked to pick cards that may describe the ‘touch point’ experienced whilst learning about the potential diagnosis of their child for the first time. They were then asked to explain their choice and use the word to tell their story in detail. Blank cards were also available to note down words that were not printed in advance. The words most frequently used are noted in the finding section of the article.

Participants were told in advance that the interview may bring back memories and evoke strong emotions, and that they could end the interview at any point. Throughout the interview the researcher attempted to sensitively check whether he fully understood what was being said and any possible alternative interpretation of the personal and intimate story that was shared. Participants were also reminded about the support they may receive during and following the interview.

At the end of the interview participants were asked if they were still willing to keep a diary and further information about this part of the study was provided. Once another consent form was signed they were helped to download the app to their personal mobile phone and encouraged to trial recording and uploading of a short video. This was followed up through a phone call and/or email to answer any further questions and to encourage participants to continue to record their video reflections. This article focuses on the analysis of the interview data only, to frame the discussion to the stories of parents learning for the first time that something may be wrong with their baby.

### Data analysis

After each interview the voice recording was transcribed and the narrative reviewed by two researchers who articulated their comments prior to jointly discussing the encounter and their observations. Each interview was exported to NVivo (Version 10, 2014), www.lumivero.com where a ‘word cloud’ was created to offer another approach to analysing the data. The comments and the visual representation of the interviews were included as additional notes for analysis. The transcript was then segmented to nodes that mirrored the words presented to the parent in a deductive way. This was done initially to open-up the data and identify the associated narrative encapsulated in those nodes. The video recordings were also transcribed and segmented according to the predefined structure of the diary, which included four set questions to be answered each time. The recording were viewed by the team and comments were again uploaded as field notes along with the transcription. Further thematic content analysis was carried out, in an inductive way to develop a theoretical scaffolding and this process was ongoing throughout the data collection phase.

## Findings

In the initial process of analysing the data and mastering the work with NVivo, we found it was helpful to quantify the frequency of use for specific emotional words. The three negative words, which were used in every interview and most often were: Scared, Anxious and Worried, followed by Confused and Vulnerable. Parents also used the words Annoyed, Frustrated, Powerless and Surprised and added a few additional words to the given list, such as Exhausted, Grieving, Uncomfortable and Stupid. These words and the stories that accompanied them, illuminated the traumatic start point, for expecting parents transitioning to a new caring role.

### Theme 1: Time – it freezes and stretches

The description of parental emotional eruption is specifically poignant when contrasting it with the silence that parents remember so vividly at the point of disclosure. The unbearable quietness that engulfs the point in time when the person attending to them becomes focused on possible foetal abnormality and the parents realising that there may be something wrong with their unborn baby.

I had my scan and the stenographer was very chatty and told me what she could see and told me, you know . . . just do some measurements . . . and then she went quiet and the nurse that was helping her was actually somebody that I know and she went quiet. She’s very chatty but she went quiet and that’s kinda when I thought uh-oh, there’s something wrong. . . – MOTHER 2

There is another waiting period when parents wait for the confirmed diagnosis, which is described using similar negative words. The days and weeks waiting for the follow-up appointment is an anxious time where parents are scared and worried.

. . .she said: ‘There could be a thousand reasons and loads of them could be nothing. It could be simple and not a problem but it had to be investigated.’ So I was worried. And I had friends asking me, ‘What’s google saying?’ [laughter] –MOTHER 4

### Theme 2: Information – When, How much and Where

During the period leading to the confirmed diagnosis parents are introduced to new terms and information about possible birth defects and level of disability. The instant medicalisation of the pregnancy is noted by the language used by some healthcare professionals. There are those who try to explain the condition and attempt to simplify it:

. . .I remember thinking like, ‘Is she a fruit bowl?’ Because I remember them saying, I know the words now but I didn’t at the time, she said the cerebrum is a banana shape and the skull is a lemon shape and it should be an orange and I’m going, ‘What’s all this fruit all about?’ [laughter] So I’m sitting quite confused going, ‘Okay, none of that makes any sense to me.’ I didn’t know what shape it was supposed to be. And she says, ‘What that’s told us straight away is that the baby has spina bifida.’ –MOTHER 4

When the diagnosis is confirmed, there is finally a degree of certainty but a lot of unknowns and many questions yet to be answered:

when we got told about the spina bifida, that’s when I thought ‘where am I supposed to go from here? What’s going to happen? Is he going to survive? Is he going to be okay? Is he going to be a lifetime of pain or?’ I didn’t know what I was supposed to expect. – MOTHER 1

when I was told, you know, I’d heard of spina bifida but I didn’t know anybody with it, I didn’t know really what it was, and, of course, the first place that you go to is Google or YouTube and that’s most definitely, which I’ve learnt now, is the worst thing to do because you get all the worst case scenarios and all the, you know, the really bad, the bad bits. –MOTHER 2

Parents are seeking information wherever they can, including through professionals, family and friends as well as by searching the Internet. Our data shows that across Scotland there is no consistency regarding practice where evidence-based, relevant and informative resources about spina bifida are offered.

### Theme 3 – Empowerment and choice

The lack of parental knowledge and the frantic pursuit for information occurs at the same time as parents grieve for the healthy baby they were expecting, and when some blame themselves for it. Such an emotionally charged time is also when parents are expected to make an informed choice about whether they wish to proceed with the pregnancy or not.

. . .get really frustrated at myself for thinking, ‘well what could I have done to stop it from happening?’ Could I have taken the folic acid beforehand? Because I didn’t know I was pregnant, so I couldn’t have stopped it. –MOTHER 1

. . . my husband was constantly asking me questions and I said, ‘I don’t know, I don’t know any more than you do, you know, that’s why we are here.’ And he was very blasé about it and very, you know, ‘It’s alright, it’s okay, it’s, you know, we’ll get through this, it’s okay’ and it wasn’t until we got taken through to that little room that I saw my husband’s face fall and he then realised no actually, there is something seriously wrong, well not seriously wrong but you know, something’s not right. –MOTHER 2

. . .but the worst thing I remember is being told, so basically baby has mylomeningocele and hydrocephalus and this is the point where mothers choose to terminate. . . . And I sunk. My heart sunk. But I remember just looking at XXX, we both looked at each other, looked back at these 2 women and I went ‘No.’ They went, ‘Right but do you understand.’ And I went, ‘No.’ but I can’t make a decision if I don’t understand anything and she says, ‘Right, well, what we are going to have to do is, we need to make an appointment for Wednesday for you to come back. –MOTHER 4

Picking up positive words enabled parents to add to their story with complementary information. The most common words used were Pleased, Fortunate, Supported and Thankful.

Yeah we were very fortunate to have XXX. Obviously, anybody is fortunate to have a child. Some people can’t have children so you should always be blessed and fortunate for the kids you do have. –MOTHER 1

But we are pleased that he doesn’t let anything stop him. You know, he’s a little boy. He has a disability but it doesn’t stop him in anyway. And we are very thankful for [him] and what he’s taught us. And we are thankful for all the support and everything we have had –MOTHER 2

The word Relieved was also noted but additional words that were often used included Happy and Brilliant. Positive words were also used describe a few excellent practitioners:

. . .so we had the scan and we had a very nice consultant, I think her name was Dr. XXX and she was just wonderful, she was so lovely, just so calm and you know I’d just got myself so worked up and had every emotion going. Then I met her. . . –MOTHER 2

I would say contact your midwife because mine was amazing and helped me and found information for me. –MOTHER 3

## Discussion

The chosen qualitative methodology and through a storytelling process, participants revisit emotions and in doing so shaped a narrative, which not only provides the participants themselves with catharsis and empowerment, but also unmask the harsh realities of care provision in Scotland. It also offers an opportunity to reflect on the process of transitioning into a caring role, on the joys of parenting and the rewards of a family life.

For all of us storytelling comes naturally and the art of telling a story can be honed in specific ways to enhance ‘meaning making’ (Burgess, 2017). Moore (2023) affirms that ‘Storytelling is a traditional method of communication that is used by the teller to entertain, build a community, and shape the beliefs, attitudes, knowledge, and behavior of listeners’ (Moore, 2023). The process of eliciting stories from research participants, as was carried out in this study, offers an element of catharsis that comes with voicing personal stories and engaging with the emotions, which (re)surfaced in an interview. There is also an empowerment to be found in the telling of personal experiences by affirming how we wish our story to be told and for our own voice to be heard, sharing experiences of ‘health and illness, tragic losses and miraculous recoveries’ (Hardy and Sumner, 2018).

The focus on specific words enabled mothers to revisit an emotional event and tell a vivid ‘origin story’, regarding the start of their caring journey. Engagement in the activity of choosing words put them in control of the way their story unfolded and offered a structure and a clear purpose to their engagement in the research. The focus on emotions and their impact in a specific and very difficult point in time was hard for participants and a few were very tearful recalling the story. It may be that mothers who had to control their emotions during the interaction, so as not to upset their child who was present, shared less impactful stories. All mothers offered important insights and showed a genuine desire to help others who may face a similar situation in the future.

The ‘wish list’ of potential interventions that may be helpful to others include a timely access to targeted support and relevant information in an accessible format. It is clear that advice, guidance and signposting to reliable resources on spina bifida is limited and reaching out to an organisation like SBH Scotland may address this need. The challenge is in ensuring both professionals and parents know about such resources. Nurses who may be present at encounters where ‘bad news’ are shared and the need for information is acute, should be able to get appropriate support themselves. This, however, is not advocating that busy nurses do even more, extend their roles or develop a dedicated team ‘in house’. Rather, front line staff should be cognisant of and seek out expertise held by specialist colleagues in third sector organisations. The needed debate about governance, information-sharing and appropriate skill mix may be led by nurses who are informed by the voices of patients as elicited in this study.

Mothers wanted effective communications in an honest, clear and detailed way so they could build trust with professionals and maximise needed therapeutic relationships. Professional conduct and a non-judgemental approach should be offered to all, irrespective of the ultimate outcome of the pregnancy, as determined by parents. Reflexive practice should be emphasised during professional training to enable practitioners to be aware of how their own beliefs may impact on the care they offer.

Taking the time to listen to parents and giving them an opportunity to review and deliberate on their options, is an essential part of offering patients’ choice. Yet, time has become a precious commodity in what is often described as a chaotic healthcare environment, especially post COVID-19. Such a reality may lead some practitioners to lose sight of the human connection and the compassionate care that frightened patients desperately need and demand.

The study had a number of limitations including the small sample size and the recruitment process, which was facilitated by a charity. The stories were told from the exclusive viewpoint of mothers, as there were no fathers who participated in the study. These mothers made a choice to keep their baby and thus offered a specific view, which may not be shared with those who made the choice of terminating their pregnancy. Carers who were not involved with SBH Scotland for a myriad of reasons were also excluded, which may also impact on the generality of findings.

## Conclusions

The Emotional Touchpoint used in this study, offered an effective way to elicit stories and highlight the long-lasting impact of trauma on expecting parents, following the diagnosis of congenital birth defects. Participatory visual narrative uses and co-creates artefacts that are ideally suited to be seen and to generate visceral impact by an observer (Hardy and Sumner, 2018). As such, results may be included in efforts to shape a different dialogue (Escobar, 2011) with policymakers to make needed changes and support carers, whose voices are often not heard. The call for future delivery of public services in Scotland, where they ‘. . .are built around people and communities, their needs. . . to build up their autonomy and resilience. . .’ (Christie Commission, 2011) is not new. This study adds weight to the call for working with people and their lived experience to help and shape the delivery of person-centred services.

Communication was highlighted as a critical skill, needed to support frightened parents, facing uncertainty about the future of their unborn child. The deafening silence that parents reported in this study, when foetal abnormalities were first detected, should be carefully considered. A scenario-based learning content, co-created with parents like the participants in this study, could be developed for Continuous Professional Development (CPD). Appreciation of the unique parental gaze and communication strategies to alleviate fears and break bad news, should become a mandatory part of nurse training.

Our study also highlighted the need for time to enable parents to consider options and assert their role in decision making. Innovative solutions should be sought for stretching time, since it is such a rare commodity in current healthcare settings. The excellent care provided by nurses and others in paediatric hospitals could be augmented by equally competent family support staff, employed by charities and not for profit organisations. Having the right governance, protocols and consent to deployment of such additional resource, could offer more time for everyone who is involved in the care process. In Scotland, the extra support offer by SBH Scotland is an example of such integrated multiagency ecosystem.

Nurses are often described as closest to their patients, due to the intimate nature of their practice. They are also noted as the agents of change in supporting transition of parents to their carer role. Considering intimacy through emotions and highlighting impact, as was done in this study, may offer nursing another way to celebrate their critical role in supporting person-centred care.

**Key points for policy, practice and/or research**

- Nurses must continue to hone needed skills and ensure effective communication supports informed decision making and supports parental choice.
- Seeking to understand the emotional impact on expecting parents, when neurological disabilities are suspected, will inform the delivery of appropriate care during and after the birth or termination of such pregnancies.
- The use of Emotional Touchpoint to elicit qualitative data was effective in highlighting specific challenges affecting nursing care practices.
- Policy to support integration between professionals working in acute settings and those in the community, including not for profit organisations, will promote family-focused and person-centred care.
